# Achieving Motor Development Milestones at the Age of Three Months May Determine, but Does Not Guarantee, Proper Further Development

**DOI:** 10.1155/2013/354218

**Published:** 2013-12-09

**Authors:** Ewa Gajewska, Magdalena Sobieska, Elżbieta Kaczmarek, Aleksandra Suwalska, Barbara Steinborn

**Affiliations:** ^1^Department of Physiotherapy, Karol Marcinkowski University of Medical Sciences, Ul. 28 Czerwca 1956 r. 135/147, 61-545 Poznan, Poland; ^2^Department and Clinic for Physiotherapy, Rheumatology and Rehabilitation, Karol Marcinkowski University of Medical Sciences, Poznan, Poland; ^3^Department of Bioinformatics and Computational Biology, Karol Marcinkowski University of Medical Sciences, Poznan, Poland; ^4^Departament of Psychiatry, Karol Marcinkowski University of Medical Sciences, Poznan, Poland; ^5^Pediatric Neurology of Developmental Age, Karol Marcinkowski University of Medical Sciences, Poznan, Poland

## Abstract

Proper motor performance at 3rd month is necessary for further motor development. The paper aims to demonstrate the reliability, sensitivity, and predictive value of an original motor performance assessment tool in comparison with the neurological assessment at 3, 6, and 9 months. Children (*n* = 123), born at term without pre- or perinatal complications, born at term with pre- or perinatal complications, or born preterm, were assessed at the age of 3, 6, and 9 months, by a neurologist and a physiotherapist. The physiotherapist evaluated 15 qualitative features typical for the age of 3 months in the prone and supine positions. The final neurological assessment determined the degree of developmental disorder. Neurological and global physiotherapeutic assessments showed a statistically significant correlation. Qualitative assessment results were very good in healthy children and decreased with worsening neurological diagnoses. Children diagnosed with cerebral palsy did not show proper qualitative features of 3 months when analyzed at 3, 6, and 9 months. Children with delayed motor development revealed minor qualitative performance impairments as early as 3 months but improved with age. Qualitative assessment at 3 months not only facilitates diagnosis of major developmental disorders but is also a good predictor of delayed motor development in children.

## 1. Introduction

Traditional assessment of motor development is based on the neuromaturational model, assuming that the pace and sequence of motor development is automatic, constitutes a reaction to the current situation, and is a manifestation of central nervous system maturity [1]. According to this concept, child development in the first year of life is genetically determined. This means that in a given month, infants develop characteristic motor functions and can generate direction-specific postural adjustments; consequently, the basic level of postural control is functionally active at this age and possibly has an innate origin [2].

Because several motor functions are common to most children at a given age, that is, they appear in a subsequent mode, a developmental assessment may be carried out. This assessment may be performed both in children at anamnestic risk, that is, with a burdened medical history, and in patients at so-called symptomatic risk, that is, without any burdened medical history, but with some features of impaired motor activity. One purpose of this assessment is to discriminate between children with motor disorders and those developing typically, even if their development is slow. Another purpose is identifying infants who may have future motor problems by assessment of their current performance. The third purpose is to assess the changes that take place over time in individual children (developmental progress and rehabilitation effect assessment) [2, 3].

At 3 months, the general movements (GMs) persist, and their quality has considerable predictive power. GM disorders occurring at 3 months indicate a 25–80% probability of future cerebral palsy [4–6]. At the same time, the 3rd month is a time of major neurodevelopmental transformation and, consequently, the time of postural control achievement [2]. Therefore, it is treated as the basis of normal future development [7, 8].

The age of 3 months is generally regarded as a time of major transition in neural function. It is possible that dissolution of the cortical subplate plays a pivotal role in the neurodevelopmental transformation occurring at this age. The subplate dissolution is accompanied by increased activity of the basal ganglia, cerebellum, and, particularly, the parietal, temporal, and occipital cortices, as demonstrated by functional neuroimaging studies [9].

The paper basEs on the following concept of quantitative and qualitative development of infants at 3, 6, and 9 months of age.

The precondition of coordinated head movements at the age of 3 months is considered normal postural activity, that is, automatic control of body posture. In the prone position, the head is precisely positioned and its isolated movements can be observed. This is possible with the infant's use of the elbows (medial condylus humeri) for support, which is one of the quantitative assessment features (YES/NO, 1/0) of global development. The infant's muscle power increases and they can more easily overcome the force of gravity, including proper head control [7, 9, 10]. Only after lifting the head can the center of gravity (the trunk-supporting point) be shifted caudally towards the pubic symphysis and the lower limbs can rest freely on the ground. The upper limbs can provide the support needed for lifting the head only in an extended posture (spine extension). The structure of this base, serving as a starting point for the development of all posture-related elements, is called a “triangle,” that is, triangular support base, which is the second element of quantitative development assessment.

In the supine position, the head is within the axis of the body, the upper limbs point towards the midline, and the lower limbs are bent up to 90 degrees in the hip and knee joints, which are all elements of the qualitative development assessment. Qualitative analysis of a child's behavior in the supine position evaluates if the upper limbs are in the intermediate position (between the internal and external rotation) with open hands [11], the full extension of the spine, and pelvis, if shoulders are in protraction, and if the feet are in an intermediate position [7]. Body balance control in the supine position is clearly visible within the pelvis and lifted lower extremities. The infant is able to control them by changing their function and position; even before the rotation of the pectoral girdle begins in the course of back-to-tummy rolling at 6 months. Another important component of motor development is axial function of the spine, acquired at the end of the 3rd month. It is achieved by means of muscle function differentiation, resulting in the appearance of support and extensor mechanisms. The first signs of lifting the body from the ground are the basis for all raising activities, subsequently appearing during motor ontogenesis, until achieving normal bipedal gait [7].

All subsequent milestones of normal motor activity are possible after learning the skills typical for 3 months, and thus, the hypothesis discussed in this paper claims that abnormal motor development in older infants is caused by failure to achieve normal quantitative and qualitative traits characteristic for 3 months of age.

According to the current literature, the next developmental milestones are 6 and 9-10 months of age [12]. At 6 months, infants develop the ability to adapt their postural activity to specific situations, are able to roll from their back to their tummy (quantitative development) around the extended longitudinal axis of the body, and, due to their ability to support their extended upper limbs and unfolded hands in pronation (quantitative development), they are preparing to achieve a quadrupedal stance [7, 12]. At the age of 9 months, infants start to develop the capacity to adapt postural adjustments in a subtle way, that is, by adapting the degree of direction-specific muscle contraction [12], and their first attempts at attaining vertical posture begin; while very immature at first, after a short time, the infant will try to stand up against furniture and walk sideways (quantitative development) [7, 12].

Considering the fact that motor development, at least involving intentional movements, depends on proper functioning of the senses, sensory-motor integration, and correct mental development, this study examined a group of children without any deficits or with motor deficits only.

Aims of the study are as follows:

1. to demonstrate the reliability, sensitivity, and predictive value of an original motor performance assessment tool;
2. to compare the concurrent validity between neurological assessment and global physiotherapeutic assessment performed at the ages of 3, 6, and 9 months;
3. to retrospectively compare (at 3, 6, and 9 months) qualitative features of motor performance at the 3rd month of life;
4. to demonstrate that correct motor performance at 6 and 9 months depends on correct quality assessment at 3 months;
5. to determine risk factors that may affect child motor development.

## 2. Material and Methods

### 2.1. Participation

The study involved primarily 140 infants. One hundred twenty-three consecutive patients without genetic or metabolic disorders or major congenital defects were qualified. There were 46 children born at term (newborn dimensions: mean head circumference of 34 ± 1 cm, mean body length of 56 ± 3 cm, mean chest circumference of 34 ± 2 cm), mean gestational age at birth of 40 ± 1 week, without pre- or perinatal complications, and with a mean birth weight of 3,527 ± 422 grams. The second group included 29 children born at term with pre- or perinatal complications. Demographic data for this group were as follows: mean head circumference at birth of 35 ± 2 cm, mean body length of 56 ± 3 cm, mean chest circumference of 34 ± 2 cm, mean gestational age at birth of 40 ± 1 week, and mean birth weight of 3,433 ± 584 grams. The third group consisted of 48 children born prematurely (newborn dimensions: mean head circumference of 31 ± 3 cm, mean body length of 49 ± 5 cm, mean chest circumference of 29 ± 4 cm), mean gestational age at birth of 34 ± 3 weeks, and mean birth weight of 1,970 ± 705 grams. Children born prematurely were examined at a corrected age [13].

The study was carried out at the Wielkopolskie Child and Youth Neurology Center in Poznań and a children's outpatient clinic in Bydgoszcz in the years of 2011–2013.

All parents consented to the study. The project was approved by the Bioethics Committee of Poznań University of Medical Sciences.

### 2.2. Procedures

All children underwent a global assessment of functional development at 3, 6, and 9 months and performed by a neurologist and a physiotherapist. At 9 months, the neurologist also assessed the degree of motor impairment and identified children with cerebral palsy (CP) or delayed motor development. According to the literature, the great majority of children with CP show its symptoms as infants or toddlers and the diagnosis of CP is made before the age of 2 years [14].

The examinations were performed independently. Both the neurologist and the physical therapist knew only that the child was born prematurely or at term but were not aware of the infant's medical history details or the parallel opinion.

The physiotherapist's assessment was based on a self-designed motor performance sheet for 3, 6, and 9 months (based on the literature, Table 5) [7, 12]. The children were observed in the supine and prone positions at 3 and 6 months, and only in the prone position at 9 months.

At 3 months, the following parameters were assessed: (1) in the supine position, the “rectangle of support,” described by the linea nuchae, spine of scapulae, and Th12, manifested by symmetrical head positioning, hands pointed towards the midline, and raising of the lower limbs above the ground; and (2) in the prone position, the “triangle of support” was described by symmetrical support on the medial epicondylus humeri and the pubic symphysis with the head raised within the body's axis.

At 6 months, the following parameters were assessed: (1) in the supine position, back-to-tummy rolling ability, and (2) in the prone position, the “rectangle of support” (support on hands and thighs).

At 9 months, the ability to stand up against furniture and walk sideways (side shuffle) was evaluated.

Possible scores of the assessments were 0 (attempt failed or only partially successful) or 1 (successful attempt). Based on the assessment, the children were classified into two groups: “developing properly” (correct) or “requiring rehabilitation” (incorrect). Physiotherapeutic assessment was always compared with neurological diagnosis (concurrent validity).

Moreover, for every child at 3 months of age, the physiotherapist examined qualitative features (using the original motor performance assessment tool) typical for 3 months of age in the prone position (15 variables) and the supine position (15 variables) (Table 5). Each feature was rated either 0 (attempt failed or only partially successful) or 1 (successful attempt). The basis for this examination was the theory that future proper motor development depends on achieving correct quality features at 3 months of age [7, 8]. The same set of quality features for the 3-month assessment was assessed in the children at 6 and 9 months as well, and the results are described in the data as “quality 3 at 6” and “quality 3 at 9,” respectively. The physiotherapeutic examination lasted about 10–15 minutes. Each assessed element had to be observed at least three to four times during the test. The presence of all 15 listed features, both in the prone and supine position, was considered normal.

The neurological development assessment was carried out according to the comprehensive neurological examination. While researching the availability of diagnostic methods, it was found that this technique is widely used, although its predictive validity for minor motor disorders is moderate at best [15]. Testing method selection may depend on the time required to complete a specific procedure, availability of other screening test resources, and the personal preference of the neurologist [16]. Neurological examination was based on the Denver Development Screening Test II (DDST II) [17], along with the evaluation of reflexes, muscle tone (hypotonia and hypertonia), and symmetry. DDST II covers all areas; however this research involved two evaluation parameters: small motor skills/precision and adaptability and movement and posture coordination/large motor skills. DDST II was chosen as it is considered an accurate method of diagnosing not only major but also minor motor disorders in term [15, 18] and preterm [19] children and because the neurologists had many years of hands-on experience with this testing method. Like most widely used and standardized tests, the DDST II has several advantages justifying its popularity. They include quick and easy application and interpretation, a comprehensible training program, the possibility of long-term assessment, psychomotor development monitoring, and disorder analysis in children with an increased risk of disorder occurrence [16, 20]. The test is designed primarily for assessing children from the first days after birth up to six years of age. Correct interpretation of results enables not only identifying retarded development but also determining the development dynamics differences. It can be successfully used at outpatient centers as a complementary method of neurological examination.

Following the examination, the neurologists classified the infants into one of three groups: “normal” (no neurological abnormalities) (group 1), “suspect” (group 2), and “abnormal” (group 3). The child was classified as “abnormal” when showing distinct neurological disorders, such as increased (hypertonia) or decreased (hypotonia) muscle tone in combination with abnormal reflexes and if they failed to complete the motor tasks for their age group in the DDST II. The children were classified as “suspect” when they exhibited mild neurological disorders such as mild problems with muscle tone control, slight reflex abnormalities, minor development asymmetry, and delayed motor development in the DDTS II.

Two physiotherapists carried out the interobserver examination independently on the same day and the results were kept blinded until final statistical analysis. Forty children were assessed via inter-observer examination. The intraobserver portion was done by comparing direct observations with the outcome of video recording analysis involving 44 infants performed at two-week intervals. The observer did not know the clinical status of the infants. Inter-observer and intra-observer reliability was examined and showed strong reliability (kappa = 0.876 and 0.871, resp.).

We considered the risk factors that may affect motor development, such as intraventricular hemorrhage (IVH) history (IVH grade none = 106 patients, I° = 8 patients, II° = 6 patients, and III° = 4 patients), Apgar score at 5 minutes (Apgar score categories 0–3 = 1 patient, 4–7 = 10 patients, and 8–10 = 114 patients), the presence of respiratory distress syndrome, intrauterine hypotrophy, and hyperbilirubinemia (based on medical records, after consulting a neurologist). At the age of 9 months neurologist pointed at children who were evolving cerebral palsy (CP). Final diagnosis was made later, at the age of 12 month.

### 2.3. Statistical Analysis

The obtained results were statistically analyzed using Statistica 10.0 (StatSoft, Inc.).

Inter-observer and intra-observer reliability was examined using the weighted kappa coefficient as a measure of inter- and intra-observer agreement. This evaluation was performed using Medcalc v. 12.4.0.0 (https://www.medcalc.org/). Logistic regression was used to compare the results of neurological and physiotherapeutic assessment. Differences between the groups classified according to neurological assessment were calculated using the Kruskal-Wallis test. The influence of risk factors were investigated using single-factor ANOVA.

In retrospective studies, the differences between groups were compared using the Wilks' lambda test.

## 3. Results

No statistical association was found between sex or prematurity and quantitative motor development.

Comparison of the developmental assessment performed by the neurologist and the assessment of motor performance carried out by the physiotherapist at 3, 6, and 9 months showed statistically significant compliance at *P* < 0.001 (Table 1).

Analysis of the results of the quantitative evaluation, prepared by the physiotherapist at 3, 6, and 9 months (Table 2), revealed that situations of motor development regress were rare. More often, motor development followed a normal course (21.8%) or a fixed scheme of improvement: more (13.7%) or less (12.9%) delayed development or serious and persisting disorders (43.5%).

Table 3 shows that, according to qualitative assessment of all patients, whether a baby was born preterm or term had no effect on motor development; therefore, further considerations were based on the neurological assessments of all children in the study group.

Results of the qualitative assessment performed by the physiotherapist were compared with the results of the neurological assessment (Table 4). It is noticeable that the qualitative assessment results for the prone and supine positions were very good in the group evaluated by the neurologist as normal, systematically decreasing along with worsening neurological performance.

**Figure 1:**
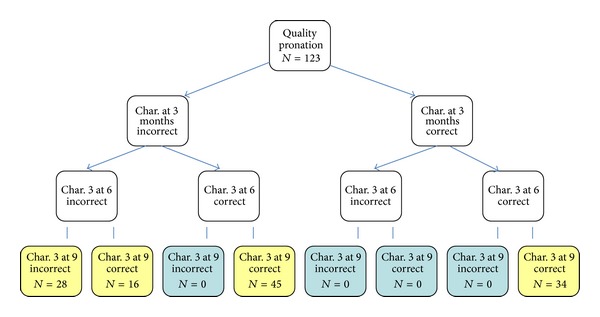
Analysis of qualitative development in the prone position at 3, 6, and 9 months.

**Figure 2:**
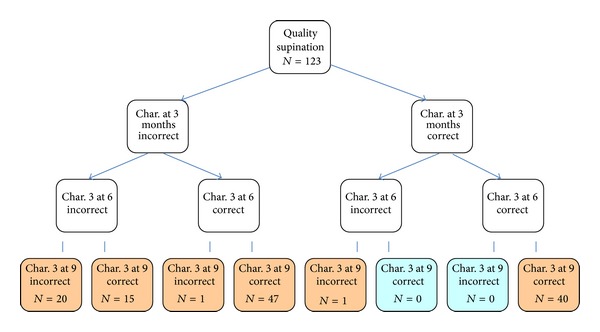
Analysis of qualitative development in the supine position at 3, 6, and 9 months.

Finally, we carried out a quantitative analysis of development for specific features of 3 months, evaluated at 3, 6, and 9 months. The analysis was performed separately for the prone (Figure 1) and supine positions (Figure 2). The study showed no developmental regress; however, no progress (incorrect-incorrect-incorrect) or delayed progress at 9 months of age (incorrect-incorrect-correct) was observed in some cases. Many children improved in the 6th or 9th month (*n* = 16 and *n* = 45, resp., in the prone position), despite initial poor performance, with more evident improvement in the supine position (*n* = 15 and *n* = 47, resp.). Lack of progress was more common in pronation.

**Figure 3:**
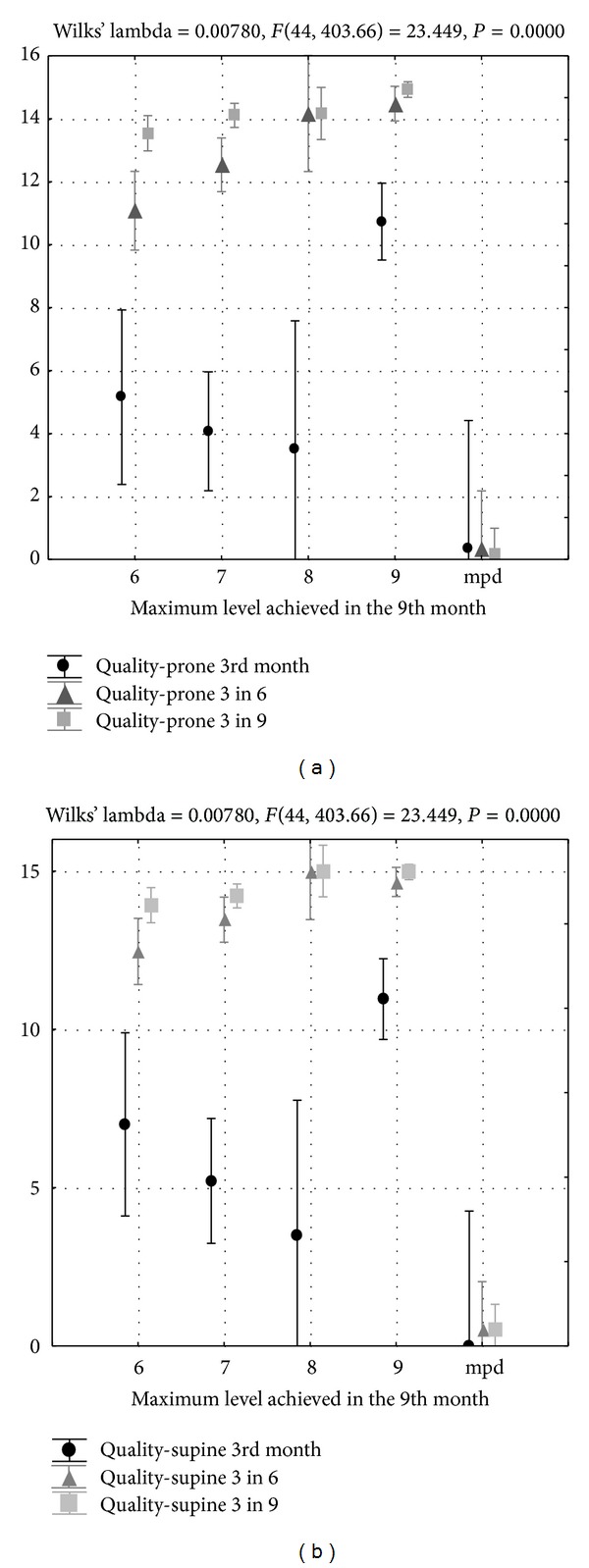
Final neurological diagnosis and qualitative assessment of motor performance at 3, 6, and 9 months.

The retrospective study shows that qualitative assessment at 3 months is a predictor of further normal abnormal motor development (Figure 3). Children diagnosed with cerebral palsy did not show the correct qualitative features of the age of 3 months when analyzed at 3, 6, and 9 months, neither in the prone nor in the supine position. However, the children neurologically classified as normal at 9 months had no qualitative disorders at 3, 6, or 9 months. The final neurological assessment also involved the children diagnosed with delayed motor development. Analysis of qualitative features proves that some minor performance disturbances decreasing with age were found in this group from the very beginning, that is, at 3 months.

Considering the gestational age, there was no correlation between preterm and term birth and the final neurological assessment results of motor performance at 9 months. Optimal development at 9 months was diagnosed in 24/49 preterm babies, 15/29 born at term but with pre- and postnatal complications, and 29/46 children born at term without pre- or perinatal complications. A developmental level of 6 months was observed in 12/49, 5/29, and 11/46 children, respectively, and cerebral palsy was diagnosed in 5/49, 2/29, and none of the full term children born without any complications.

We analyzed risk factors that may affect quantitative and qualitative motor performance in the subsequent months. The following factors were considered: results of a brain ultrasound (*n* = 55 normal, *n* = 46 abnormal), IVH event (*n* = 18), respiratory distress syndrome (*n* = 15), SGA (*n* = 8), hyperbilirubinemia (*n* = 22), and Apgar score at 5 minutes. It was found that only intraventricular hemorrhage (IVH) episodes were important for the qualitative assessment in prone position at *P* = 0.012, *χ* ^2^ = 14.676, odds ratio: quality at 3 months = 0.949, quality 3 at 6 months = 1.989, and quality 3 at 9 months = 1.023, and in the supine position: quality at 3 months = 1.749, quality 3 at 6 months = 0.849, and quality 3 at 9 months = 2.603. Considering the severity of intraventricular bleeding, the final diagnosis of IVH children revealed: IVH III°-one child developing normally, one retarded, and two diagnosed with cerebral palsy; IVH II°-five cases of developmental retardation, one child diagnosed with cerebral palsy; IVH I°-four children developing normally and four retarded.

## 4. Discussion

Infant motor development is often interpreted as the mastery of increasingly complicated reflexes. However, it is well known that maturation is determined not only genetically (reflexes) but also environmentally, through developmental processes such as synaptogenesis, depending on the quality and type of stimuli present. Higher nerve centers will take control over lower nerve centers, that is, the individual spinal cord reflex system. This process manifests by the gradual loss of automatic behaviors and primitive reflexes.

Prechtl claims that fetal movement patterns can be considered as clearly genetically determined features of the nervous system, depending on the stage of their development. He compares the entire repertoire of fetal activity, observed from 9 weeks of gestation to postnatal movement patterns. He also emphasizes the continuity of motor development from the fetal into the infancy period [5, 21], whereas the process of motor pattern selection depends on the organism functioning in a changing environment.

Determining the range of motor abilities during the first week postnatal is as important as the detection of pathological features. Both elements are crucial for an early diagnosis of the infant's neurological status. Beginning in the first days and weeks of life, basic reflexes tend to be gradually replaced by intentional, precise, and progressively more conscious activities. The main factor of this evolution is controlling the position of the head and trunk, which begins with a clear definition of the body axis, and this process takes place at the age of 3 months.

Developmental changes in an infant can be analyzed using standardized tests. They are expected to be useful and easily applicable to differentiate patients with delayed and normal development and to evaluate physiotherapy results, that is, to meet the concept of minimal clinically important change [18, 21–23].

One of the aims of the study was to select, based on available literature, quality features that are considered important in ideal motor activity but also that form the basis for proper further development. As confirmed by the literature, features of 3 months constitute such a base [7, 8].

First, we were able to prove that global neurological and physiotherapeutic assessment are convergent, even though they are based on different assumptions. This shows to what extent motor disorders are associated with global developmental assessment. Neurological examination also reveals the motor impairments that are defined in detail only during qualitative physiotherapeutic assessment. It is worth noting that increasing compliance of neurological and physiotherapeutic assessment indicates that, at an early stage, a neurologist classifies children with minor impairments as normal, while the physiotherapist already suggests the need for rehabilitation. Over time, when disorders become more visible, those two assessments are increasingly more similar.

It should also be noted that this convergence is clearly visible when a detailed qualitative assessment is considered. The neurologist diagnosed as normal those infants who according to the physiotherapist achieved all the required quantitative features, while those with exacerbating deficits were qualified as less developed. Thus, even if the Denver test assessment is considered imprecise, an experienced neurologist assesses the same motor activity elements as the physiotherapist, even if they are not clearly defined.

We next analyzed the global motor performance assessed by the physiotherapist at 3, 6, and 9 months. Most of the infants developed according to a certain pattern: they manifested good motor development throughout the study, they improved over time, or they were clearly abnormal from the beginning. There was also a small group of children whose initially normal motor performance deteriorated, or improved slightly and then worsened again. For example, three children developed normally until 6 months of age, and at 9 months, their performance was impaired (correct-correct-incorrect scheme), and other three children regressed at 6 months and later improved (correct-incorrect-correct). The authors are not able to prove to what extent additional factors (infection, trauma, and hospital stay) may have contributed to this arrested development, but it is worth noting that this pattern is different from the one described for IVH-related CNS damage.

Qualitative features of the 3rd month were assessed at 3, 6, and 9 months in the prone and supine positions. The study showed that if the qualitative assessment for the 3rd month in the prone position, performed at 3 months of age, was normal; it was also correct in up to 87% of infants at 9 months. However, if the qualitative assessment at 3 months was incorrect and improved until 6 months, it did not change at 9 months of age. Similarly, when the qualitative assessment was incorrect at 3 months and did not improve at 6 months, it did not change and was still incorrect at 9 months.

However, the qualitative assessment in the supine position showed a slightly different pattern. If it was incorrect at 3 months, it could be improved at up to 9 months in 18% of the patients, and if performance at 3 months was correct, it remained constant and correct at 6 months, and in 98% of the patients, at 9 months. It seems that the assessment of qualitative features is more sensitive in the prone position, reflecting a more demanding antigravity postural control (greater involvement of antigravity muscles such as the pectoralis major and subscapularis) [7].

The retrospective study shows that qualitative assessment at 3 months is a predictor of further correct or impaired motor development (Figure 3) and reflects the changes related to the developmental transformations of the infant brain [24]. In children diagnosed with cerebral palsy, qualitative performance was incorrect from the beginning, but it is very important that the qualitative assessment has proved to be a good predictor for children with delayed motor development, that is, those who develop slower but can eventually achieve normal developmental status. Therefore, qualitative analysis of the infant may help to predict not only major disorders but also minor neurological dysfunctions, assisting in early planning and initiation of a targeted therapy.

It is also worth emphasizing that no link was found between premature or full term birth with or without pre- or perinatal complications and motor development at 9 months. The number of children classified as preterm or full term that did not reach the maximum development level at 9 months was similar. The condition of prematurity is not a risk factor, and neither full term nor uncomplicated delivery will guarantee normal motor development.

Risk factor assessment is an important element of child development observation. Environmental, genetic, biological [25–27], social, and demographic [28] factors may increase the risk of developmental delays, and therefore, the children exposed to those factors more often require neurological assessment and observation. The present study discussed only biological factors. For most of them, no adverse effects on infant motor development were found. The only significant factor was the already mentioned intraventricular hemorrhage (IVH) events [29]. When they were analyzed collectively or according to IVH seriousness, it became apparent that at least 75–80% were associated with persisting disorders of motor development (incorrect-incorrect-incorrect scheme), especially grade IV of IVH, which is consistent with previous observations and research. The researchers claim that grade III of IVH is associated with future psychomotor development abnormalities in about 35% of children, and for grade IV, the risk increases to approximately 90% [30, 31].

## 5. Conclusions

1. The quality assessment of motor performance was shown to be a reliable and sensitive predictor of disorders with a high predictive value when compared to neurological assessment.
2. Quality features of 3 months are good predictors of further development.
3. Qualitative assessment at 3 months of age not only facilitates the diagnosis of major developmental disorders but also is a good predictor for children with delayed motor development.
4. Intraventricular hemorrhage may affect infant motor development.

## Figures and Tables

**Table 1:** Neurological and physiotherapeutic assessment at 3, 6, and 9 months of life.

Neurological assessment	Physiotherapeutic assessment		
Three-month-old children	Developing properly	Requiring rehabilitation	Total
Normal (no neurological abnormalities)	33 (9, 7, 17)	5 (3, 2, 0)	38
Suspect	1 (0, 1, 0)	19 (8, 5, 6)	20
Abnormal	0 (0, 0, 0)	65 (28, 14, 23)	65
Total	**34**	**89**	**123**
	Odds ratio = 113.82; *χ* ^2^ = 117.70, *P* < 0.001		
Six-month-old children	Developing properly	Requiring rehabilitation	Total
Normal (no neurological abnormalities)	48 (18, 10, 20)	4 (0, 2, 2)	52
Suspect	0 (0, 0, 0)	24 (7, 7, 10)	24
Abnormal	0 (0, 0, 0)	47 (24, 9, 14)	47
Total	**48**	**75**	**123**
	Odds ratio = 194.49; *χ* ^2^ = 136.33, *P* < 0.001		
Nine-month-old children	Developing properly	Requiring rehabilitation	Total
Normal (no neurological abnormalities)	63 (21, 14, 28)	5 (1, 2, 2)	68
Suspect	0 (0, 0, 0)	31 (11, 8, 12)	31
Abnormal	0 (0, 0, 0)	24 (16, 5, 3)	24
Total	**63**	**60**	**123**
	Odds ratio = 104.60; *χ* ^2^ = 136.14, *P* < 0.001		

The number of children in parentheses denotes infants born prematurely, born at term but with pre- or perinatal complications, and born at term without pre- or perinatal complications.

**Table 2:** Global (quantitative) assessment of development at 3, 6, and 9 months, performed by the physiotherapist.

Results of the global (quantitative) physiotherapeutic assessment	Number of patients	Percentage
Incorrect-correct-incorrect	2	1.6
Correct-incorrect-incorrect	1	0.8
Correct-incorrect-correct	3	2.4
Correct-correct-incorrect	3	2.4
Correct-correct-correct	**27**	**21.8**
Incorrect-incorrect-incorrect	**54**	**43.5**
Incorrect-incorrect-correct	**17**	**13.7**
Incorrect-correct-correct	**16**	**12.9**

	*N* = 123	100%

Bold font refers to total numbers.

**Table 3:** Qualitative assessment score (maximum of 15) at 3 months, performed by a physiotherapist; data reported according to gestational age and neurological assessment. Results are given as median (quartiles 25–75%).

	Normal (no neurological abnormalities)	Suspect	Abnormal
Born prematurely			
Quality in position: prone, supine	*N* = 12	*N* = 9	*N* = 27
Prone	15 (13–15)	8 (0–12)	0 (0–11)
Supine	15 (9–15)	12 (0–15)	0 (0–11)
Born at term w/pre- or perinatal complications			
Quality in position: prone, supine	*N* = 9	*N* = 6	*N* = 14
Prone	15 (10–15)	9 (7–15)	2 (0–11)
Supine	15 (15-15)	11 (7–15)	5 (0–15)
Born at term w/o pre- or perinatal complications			
Quality in position: prone, supine	*N* = 17	*N* = 6	*N* = 23
Prone	15 (15-15)	11 (9–13)	2 (0–11)
Supine	15 (15-15)	12 (11–13)	2 (0–11)

**Table 4:** Qualitative assessment scores (maximum score of 15) in infants at 3, 6, and 9 months in prone and supine position, reported according to neurological assessment. Results are given as median (quartiles 25–75%).

Characteristics	Neurological assessment at 3 months—normal (no neurological abnormalities) (*n* = 38)	Neurological assessment at 3 months—suspect (*n* = 20)	Neurological assessment at 3 months—abnormal (*n* = 65)	Significance of differences, *P* =
Quality prone position—3 months	15 (15-15)	9 (8–12)	0 (0–6)	*P* < 0.0001
Quality supine position—3 months	15 (15-15)	12 (9–13)	2 (0–6)	*P* < 0.0001

	Neurological assessment at 6 months—normal (no neurological abnormalities) (*n* = 52)	Neurological assessment at 6 months—suspect (*n* = 24)	Neurological assessment at 6 months—abnormal (*n* = 47)	

Quality prone position—3 months	15 (12–15)	7 (0–11)	0 (0–6)	*P* < 0.0001
Quality supine position—3 months	15 (12–15)	8 (0–11)	0 (0–6)	*P* < 0.0001
Quality prone position—3 at 6	15 (15-15)	15 (13–15)	12 (8–14)	*P* < 0.0001
Quality supine position—3 at 6	15 (15-15)	15 (13–15)	13 (11–15)	*P* < 0.0001

	Neurological assessment at 9 months—normal (no neurological abnormalities) (*n* = 67)	Neurological assessment at 9 months—suspect (*n* = 32)	Neurological assessment at 9 months—abnormal (*n* = 24)	

Quality prone position—3 months	13 (8–15)	6 (0–9)	0 (0–3)	*P* < 0.0001
Quality supine position—3 months	15 (8–15)	6 (0–11)	0 (0–5)	*P* < 0.0001
Quality prone position—3 at 6	15 (15-15)	15 (12–15)	11 (0–13)	*P* < 0.0001
Quality supine position—3 at 6	15 (15-15)	15 (13–15)	12 (0–14)	*P* < 0.0001
Quality prone position—3 at 9	15 (15-15)	15 (13–15)	13 (3–15)	*P* < 0.0001
Quality supine position—3 at 9	15 (15-15)	15 (15-15)	13 (5–15)	*P* < 0.0001

**Table:** (a)

Sum of the qualitative characteristics	Yes	No
Head:		
(1) Isolated head rotation		
Shoulders and upper limbs:		
(2) Arm in front, forearm in intermediate position, elbow outside of the line of the shoulder (R)		
(3) Arm in front, forearm in intermediate position, elbow outside of the line of the shoulder (L)		
(4) Palm loosely open (R)		
(5) Palm loosely open (L)		
(6) Thumb outside (R)		
(7) Thumb outside (L)		
Spine and pelvis		
(8) Spinal cord segmentally in extension		
(9) Scapula situated in medial position (R)		
(10) Scapula situated in medial position (L)		
(11) Pelvis in intermediate position		
Lower limbs		
(12) Situated loosely on the substrate (R)		
(13) Situated loosely on the substrate (L)		
(14) Foot in intermediate position (R)		
(15) Foot in intermediate position (L)		

Maximum of 15 points for qualitative characteristics.

**Table:** (b)

Sum of the qualitative characteristics	Yes	No
(1) Head symmetry		
(2) Spinal cord in extension		
(3) Shoulder in balance between external and internal rotation (R)		
(4) Shoulder in balance between external and internal rotation (L)		
(5) Wrist in intermediate position (R)		
(6) Wrist in intermediate position (L)		
(7) Thumb outside (R)		
(8) Thumb outside (L)		
(9) Palm in intermediate position (R)		
(10) Palm in intermediate position (L)		
(11) Pelvis extended (no anteversion and retroversion)		
(12) Lower limb situated in moderate external rotation (R)		
(13) Lower limb situated in moderate external rotation (L)		
(14) Lower limb bent at a right angle at hip and knee joints, foot in intermediate position—lifting above the ground (R)		
(15) Lower limb bent at a right angle at hip and knee joints, foot in intermediate position—lifting above the ground (L)		

Maximum of 15 points for qualitative characteristics.
